# Consistency and replicability of a pharmacist-led intervention for asthma patients: Italian Medicines Use Review (I-MUR)

**DOI:** 10.1017/S1463423618000580

**Published:** 2018-09-13

**Authors:** Andrea Manfrin, Janet Krska

**Affiliations:** 1 Senior Lecturer in Pharmacy Practice, Sussex Pharmacy, School of Life Sciences, University of Sussex, Falmer, Brighton, UK; 2 Professor of Clinical and Professional Practice, Medway School of Pharmacy, Universities of Greenwich and Kent at Medway, Anson Building, Central Avenue, Chatham Maritime, Chatham, Kent, UK

**Keywords:** asthma, community pharmacy, consistency, Medicines Use Review, replicability

## Abstract

**Aim:**

This study aimed to assess the consistency and replicability of these process measures during provision of the Italian Medicines Use Review (I-MUR).

**Background:**

Medication review is a common intervention provided by community pharmacists in many countries, but with little evidence of consistency and replicability. The I-MUR utilised a standardised question template in two separate large-scale studies. The template facilitated pharmacists in recording medicines and problems reported by patients, the pharmaceutical care issues (PCIs) they found and actions they took to improve medicines use.

**Methods:**

Community pharmacists from four cities and across 15 regions were involved in the two studies. Patients included were adults with asthma. Medicines use, adherence, asthma problems, PCIs and actions taken by pharmacists were compared across studies to assess consistency and replicability of I-MUR.

**Findings:**

The total number of pharmacists and patients completing the studies was 275 and 1711, respectively. No statistically significant differences were found between the studies in the following domains: patients’ demographic, patients’ perceived problems, adherence, asthma medicines used and healthy living advice provided by pharmacists. The proportion of patients in which pharmacists identified PCIs was similar across both studies. There were differences only in the incidence of non-steroidal anti-inflammatory drug use, the frequency of potential drug-disease interactions and in the types of advice given to patients and GPs.

**Conclusions:**

The use of a standardised template for the I-MUR may have contributed to a degree of consistency in the issues found, which suggests this intervention could have good replicability.

## Background

Medication review is a cognitive pharmaceutical service (CPS) (Benrimoj *et al*., 2010) provided by community pharmacists in a range of countries (Barbanel *et al*., 2003; Emmerton *et al*., 2003; McLean *et al*., 2003; Bunting and Cranor, 2006; Mehuys *et al*., 2008; García-Cárdenas *et al*., 2013). One of the earliest funded services is the Medicines Use Review (MUR) service introduced in England in 2005, for which there is relatively little evidence to support either its effectiveness (Wright, 2016) or cost-effectiveness (Centre for Policy on Ageing, 2014). A recent systematic review and meta-analysis of randomised controlled trials of medication review suggested that an isolated medication review has minimal effect on clinical outcomes, no effect on quality of life and lacks evidence of economic outcomes, although studies have shown a decrease in the number of drug-related problems (DRP), more changes in medication, more drugs with dosage decrease and a greater decrease or smaller increase of the number of drugs used (Huiskes *et al*., 2017).

Studies which have focussed on specific conditions, however, have consistently shown positive outcomes. For example, in patients with asthma, studies in several countries have shown that pharmacists can identify problems with medicines and intervene to improve outcomes (García-Cárdenas *et al*., 2016), and that there is a need for such intervention. A study in Denmark (Herborg *et al*., 2001) and more recent studies conducted in India (Gajanan *et al*., 2015) and Vietnam (Nhu Nguyen *et al*., 2018) found that patients had poor knowledge of asthma. A study in Germany found that the most common advice given to asthmatic patients was education about their medicines (Schulz *et al*., 2001). Although many well-designed studies (Narhi *et al*., 2000; Cordina *et al*., 2001; Herborg *et al*., 2001; Schulz *et al*., 2001; Barbanel *et al*., 2003; Emmerton *et al*., 2003; McLean *et al*., 2003; Saini *et al*., 2004; Mangiapane *et al*., 2005; Bunting and Cranor, 2006; Haahtela *et al*., 2006; Armour *et al*., 2007; Mehuys *et al*., 2008; García-Cárdenas *et al*., 2013) have been carried out in asthma, very few provided evidence of effectiveness (Armour *et al*., 2007; García-Cárdenas *et al*., 2013). A study conducted by Armour *et al*. (2012) assessed the feasibility and sustainability of a CPS for patients with asthma, but did not assess consistency and replicability. According to the Oxford Dictionary, consistency is the quality of achieving a level of performance, which does not vary greatly in quality over time; replicability represents the ability of a scientific experiment or trial to be repeated to obtain a consistent result.

In Italy, although the Government (Legge 69/2009 e D.LGS 153/2009) approved the provision of CPS in 2009, no services are delivered by community pharmacies. In contrast to many other countries, Italian community pharmacists are not permitted to keep patient records of medication dispensed, hence reviewing medicines is less feasible. Moreover, Italian pharmacists do not receive training in clinical pharmacy as part of their undergraduate training, and postgraduate training in this area is also not widely available. The Italian Pharmacists’ Federation (FOFI), recognising the extent to which Italian pharmacy had failed to move forward with other countries in developing CPS, identified the need for different types of evidence to be obtained locally before any service could be commissioned. FOFI collaborated with academic researchers to develop a programme of studies to fulfil this need. The Italian Medicines Use Review (I-MUR) project began in 2010 and took five years to complete. The I-MUR developed was a structured approach to medication review, based on the English service but with several key differences. Based on evidence of the benefits of CPS in asthma, this condition was selected for the programme and the I-MUR designed specifically for patients with asthma. It was constructed using mostly closed questions to enable the community pharmacists providing the intervention to easily gather the data essential for demonstrating the type of evidence which could ultimately support the continuation of such services.

The I-MUR programme involved three phases: Phase 1 (intervention testing), 2 (evaluation) and 3 (cluster randomised controlled trial). Phases 1 and 3, in which pharmacists delivered the I-MUR, were conducted in 2012–2013 and 2014–2015, respectively. Phase 1 was a non-randomised study with no follow-up, conducted in four cities in Northern Italy which identified the potential for benefit. This study aimed to determine whether pharmacists were able to undertake the process of completing an I-MUR with asthmatic patients and upload data onto a web platform. Both pharmacists and patients involved in Phase 1 were excluded from Phase 3, which was a cluster randomised control trial including an economic analysis, conducted in 15 regions across the whole of Italy. Phase 3, reported elsewhere (Manfrin *et al*., 2017), demonstrated that the I-MUR was both effective and cost-effective. Results from the evaluation (Phase 2) which obtained the views of pharmacists, patients and GPs on the I-MUR service provided during Phase 1 have also been published (Manfrin and Krska, 2018).

However, for a service to be commissioned and funded, there should be demonstrable capacity to benefit (need), consistency and replicability of the structures and processes which contribute to clinical effectiveness should be assured (Donabedian, 1980). As no previous studies had been undertaken in Italy of CPS, the collection of process data was an important aspect of evaluating delivery of the I-MUR intervention. For an intervention to become standard practice, a degree of consistency is needed and for a particular outcome to be achieved, the structures and processes should be similar. Previous studies have shown that delivery of a medication review intervention varies between individual pharmacists (Krska and Avery, 2008; Hinchliffe, 2011). We therefore examined key process measures which could contribute to the effectiveness of the I-MUR intervention to assess the potential consistency of delivery, in the absence of formal fidelity testing, which is regarded as the extent to which a test duplicates the actual conditions or task performed; the closer the match, the higher the fidelity of the test. We also assessed the potential need for the service (potential to benefit) by review of the problems patients reported with their asthma and their medicines.

## Aims

The aims of this study were to assess the consistency and replicability of I-MUR by:

- comparing the demographics, medications (active ingredients) used, self-reported adherence and problems with asthma and medicines of patients involved in Phases 1 and 3 of the I-MUR programme;
- quantifying and comparing the types of pharmaceutical care issues (PCIs) and actions taken by pharmacists during the provision of I-MUR in both phases.

## Methods

### Selection and recruitment of pharmacies, pharmacists and patients

Local pharmacy organisations in each of the four regions (Phase 1) and 15 regions (Phase 3) invited all community pharmacists to participate (using phone calls and emails). For those who expressed interest in the study, a selection process was undertaken to ensure that pharmacists all met pre-specified inclusion criteria, which included a private room, internet connection and provision of some services beyond dispensing from their pharmacy. Patients were recruited by the pharmacists on the basis of either a diagnosis or prescription of medicines for asthma. Further details of all inclusion and exclusion criteria for pharmacies, pharmacists and patients have been published elsewhere (Manfrin *et al*., 2015).

### The I-MUR intervention

The I-MUR involves a face-to-face consultation between pharmacist and patient in a private room. The I-MUR was generally based on the English MUR template, but a new more systematic, structured interview template was developed, specifically for asthma patients, which used closed questions allowing quantitative data to be gathered easily. Questions in the template covered: asthma symptoms, medicines used (active ingredients), problems and adherence. The first version of I-MUR was developed by A.M. for Phase 1, and was validated by eight Italian non-participating community pharmacists. The results of the evaluation (Phase 2) provided the opportunity to add two questions to the I-MUR instrument before using it in Phase 3. All pharmacists in both Phases (1 and 3) received training regarding asthma physiopathology and clinical pharmacology from respiratory physicians (1 h). A.M. provided 3 h of training in Phase 1 and 4 h in Phase 3. The difference in the length of training was due to higher complexity of Phase 3. The training provided was in pharmaceutical care, in particular how to identify PCIs, which could have an impact on the use of medicines and/or asthma control and to provide appropriate advice to patients and recommendations to their GPs. The latter were based on the individual pharmacists’ clinical judgements using all the data gathered during the I-MUR. Pharmacists were also trained in the use of the I-MUR template to gather data and in uploading it onto the web platform. Use of this system enabled pharmacists to enter patient-level data in Italy, which was then downloaded for analysis in the United Kingdom, thus avoiding the complexities of paper-based data collection. In order to provide the I-MUR, the pharmacists had to request details of all medicines patients were using, due to the lack of patient medication records. For each patient, pharmacists recorded: medicines used, responses to closed questions on asthma, problems and adherence, PCIs they identified and actions/recommendations they made.

### Data analysis

Medications (active ingredients) were classified using the World Health Organization (WHO) anatomic, therapeutic, chemical (ATC) classification system. The frequency of use of three major drug classes, non-steroidal anti-inflammatory drugs (NSAIDs), ace-inhibitors (ACE-Is) and *β*-blockers not recommended in asthmatic patients was determined. The number of PCIs identified and actions taken during the I-MUR service provision were classified by the pharmacists using the methods of Krska *et al*. (2002) before adding to the web platform. This classification system, which has been used in medication review studies in England (Krska and Avery, 2008), was deemed sufficiently simple for use by the Italian pharmacists.

Comparisons between the data obtained from Phases 1 and 3 were made using three non-parametric techniques. (*χ* ^2^) for independence (Pearson’s *χ* ^2^) was used when comparing the relationships between two categorical variables, and when each of these variables could have had more than two or more categories. Fisher’s exact test for independence was used when the frequency was below five. *χ* ^2^ test for goodness-of-fit was used with categorical variable when comparing the proportion of cases from a sample with hypothesised of those obtained previously from a comparison population. Owing to the number of hypotheses tested (53 items), the Bonferroni correction was adopted, as suggested by Goldman (2008), resulting in a level of significance of *P*=0.0009. The analysis was conducted using Microsoft Excel version 2016 and SPSS version 24.

## Findings

### Demographic details

In Phase 1, four Italian regions and four specific locations (towns) were involved: Piemonte (Torino), Toscana (Pistoia), Lombardia (Brescia) and Veneto (Treviso). The number of pharmacists enrolled in Phase 1 was 74 and they recruited 895 patients. Phase 3 was powered to detect a clinically significant difference in ATC score, using a large number of pharmacists across Italy, to minimise the effect of inter-pharmacist variation on the primary outcome. Therefore, 201 pharmacists and 816 patients completed Phase 3. The number of regions involved in this phase was 15: Trentino Alto Adige, Lombardia, Sicilia, Puglia, Sardegna, Piemonte, Valle d’Aosta, Veneto, Friuli Venezia Giulia, Toscana, Emilia Romagna, Marche, Abruzzo, Lazio and Campania.

Patients recruited to both studies showed similar gender and age distributions, spanning a large range of ages (Table 1).

**Table 1:** Patients’ demographic and asthma active ingredients

	Phase 1		Phase 3		
	*n*	%	*n*	%	*P*-value
Gender					0.0237
Female	491	54.9	480	58.8	
Male	404	45.1	336	41.2	
Total	895		816		
Age range					0.0060
18–40	170	19.1	195	23.9	
41–50	128	14.3	156	19.1	
51–60	146	16.4	153	18.8	
61–70	185	20.7	159	19.5	
Over 70	263	29.5	153	18.8	
Total number of patients	892		816		
Missing	3				
Active ingredients					
Corticosteroids (eg, beclomethasone)	660	73.7	638	78.2	0.0088
*β*-2 agonists long-acting (eg, salmeterol)	637	71.2	617	75.6	0.0102
*β*-2 agonists short-acting (eg, salbutamol)	488	54.5	398	48.8	0.0374
Antimuscarinic bronchodilators (eg, ipratropium)	216	24.1	142	17.4	0.0012
Leukotriene receptor antagonists (eg, montelukast)	146	16.3	231	28.3	0.0010
Theophylline	28	3.1	29	3.6	0.1407
Cromoglicate and nedocromil	4	0.4	14	1.7	0.0153^b^
Omalizumab (only prescribed by special centres)	1	0.1	6	0.7	0.0589^b^
Total number of patients	895		816		

*χ* ^2^ with Bonferroni adjustment 0.05/53 0.0009

a Statistical significant difference.

b Fisher’s exact test with Bonferroni adjustment.

### Medicines used (as active ingredients)

The median number of all active ingredients used by patients before receiving the I-MUR was 5.0, in both Phases 1 and 3. No statistically significant differences were found across the two populations in the active ingredients used for treating asthma (Table 1), with the most common active ingredients being corticosteroids (73.7%, Phase 1; 78.2%, Phase 2) and long-acting *β*-2 agonists (71.2%, Phase 1; 75.6%, Phase 2), whereas the least common was omalizumab (0.1%, Phase 1; 0.7% Phase 2). Overall, the proportion of patients using active ingredients considered to be inappropriate in asthmatic patients (ACE-I, *β*-blockers and NSAIDs) was slightly higher in Phase 3 compared with Phase 1, but the difference was not statistically significant (Table 2).

**Table 2:** Pharmaceutical care issues (PCIs) and active ingredients

	Phase 1		Phase 3		
	*n*	%	*n*	%	*P*-value
Type of PCIs					
Education required	259	28.9	245	30	0.1617
Monitoring issues	216	24.1	165	20.2	0.0554
Discrepancy between dose prescribed and drug used	156	17.4	159	19.5	0.0613
Potential ineffective therapy	146	16.3	122	15	0.2055
Potential/actual compliance/adherence	128	14.3	112	13.7	0.2271
Inappropriate dose regimen	116	13	90	11	0.1252
Potential/suspected adverse drug reaction	101	11.3	122	15	0.0044
Potential drug-disease interaction	82	9.2	115	14.1	0.0002^a^
Inappropriate duration of therapy	53	5.9	42	5.1	0.1674
Untreated indication for therapy	40	4.5	32	3.9	0.1739
Repeat prescription no longer required	22	2.5	23	2.8	0.144
Drug use with no indication	19	2.1	29	3.6	0.0185
Others	2	0.2	0	0	0.5007^b^
Total number of patients	895		816		
Active ingredients					
ACE-I	114	12.7	90	11	0.1452
*β*-Blockers	64	7.2	68	8.3	0.0838
NSAID	261	29.2	340	41.7	<0.0001^a^
Total number of patients	895		816		

*χ* ^2^ with Bonferroni adjustment 0.05/53 0.0009

ACE-I=angiotensin-converting-enzyme inhibitors; NSAID=non-steroidal anti-inflammatory.

a Statistical significant difference.

b Fisher’s exact test with Bonferroni adjustment.

### Self-reported adherence and problems with medicines

Patients’ self-reported adherence to medications was low in both phases: 51.4% (*n*=460) in Phase 1 and 45.8% (*n*=374) in Phase 3 (*P*=0.0214; not statistically significant). Around a quarter of patients in both studies perceived they had problems with their asthma medications, whereas around three-quarters considered they knew how to use them, considered they were working and were effective (Table 3).

**Table 3:** Patients’ perceptions of their medications

	Phase 1		Phase 3		
	*n*	%	*n*	%	*P*-value
How patients were getting on with their medications					0.8300
Did not have problems	640	72.2	573	70.9	
Had some problems	227	25.6	217	26.9	
Had lots of problems	19	2.1	18	2.2	
Total number of patients	886		808		
Missing	9		8		
Did patients have enough knowledge and understanding about how to take their medications?					0.4700
Knew how to take the medications fully	689	77.8	611	75.2	
Knew how to take the medications partially	187	21.1	191	23.5	
Did not know how to take the medications at all	10	1.1	10	1.2	
Total number of patients	886		812		
Missing	9		4		
What did patients think about their medications?					0.4700
All were working	659	74.7	629	77.7	
Some were working	180	20.4	149	18.4	
None were working	6	0.7	3	0.4	
Did not know	37	4.2	29	3.6	
Total number of patients	882		810		
Missing	13		6		
Did patients think their medications were effective as they were expecting?					0.0020
Yes	667	75.6	627	77.2	
No	130	14.7	80	9.9	
Did not know	85	9.6	105	12.9	
Total number of patients	882		812		
Missing	13		4		

*χ* ^2^ with Bonferroni adjustment. 0.05/53 0.0009

a Statistical significant difference.

b Fisher’s exact test with Bonferroni adjustment.

### PCI identified and actions taken

Despite patients perceiving they knew how to use medicines, in Phase 1 pharmacists identified at least one PCI in 543 (60.7%) patients, a mean of 2.4 per patient among those with a PCI. In Phase 3, 64.6% of patients (527/816) had a PCI with the mean being 2.5 per patient. The three most common PCIs in both phases were the same: education required, monitoring issues and discrepancy between dose prescribed and drug used (Table 2).

In both Phases, the most common type of action taken by pharmacists was to provide drug information to patients. Overall, there were very few differences between the two studies in the types of actions taken by pharmacists to improve medicines use (Table 4). The frequency with which advice regarding healthy living was provided also showed no differences between the phases. In particular, pharmacists provided advice to stop or reduce smoking to 23 and 25% of patients in Phases 1 and 3, respectively. The need to carry out monitoring was the most frequent type of advice given to GPs in both phases, but was slightly more common in Phase 3, as was the recommendation to change a drug and other advice.

**Table 4:** Frequency of advice provided by pharmacists to patients and general practitioners

	Phase 1		Phase 3		
	*n*	%	*n*	%	*P*-value
Advice given to patients					
Drug information provided	659	73.6	585	71.7	0.4560
Consult GP	355	39.7	355	43.5	0.0226
Change method of administration	119	13.3	192	23.5	<0.0001^a^
Change dose	86	9.6	94	11.5	0.0443
Change time of administration	54	6.0	64	7.8	0.0321
Stop non-prescription drugs	38	4.2	71	8.7	<0.0001^a^
Other	10	1.1	88	10.8	<0.0001^a^
Total number of patients	895		816		
Healthy living advice to patients					
Physical activity	344	38.4	332	40.7	0.0884
Diet and nutrition	329	36.8	368	45.1	0.0060
Weight management	236	26.4	234	28.7	0.0660
Smoking	204	22.8	203	24.9	0.0719
Alcohol	79	8.8	61	7.5	0.1397
Other	16	1.8	26	3.2	0.0159
Sexual health	11	1.2	21	2.6	0.0106
Total number of patients	895		816		
Advice given to GPs					
Carry out monitoring	471	52.6	491	60.2	0.0002^a^
Provide compliance/adherence aid	261	29.2	268	32.8	0.0197
Change dose	72	8.0	82	10.0	0.0330
Add a drug	71	7.9	46	5.6	0.0374
Change drug	53	5.9	91	11.2	<0.0001^a^
Change computer record	33	3.7	21	2.6	0.0802
Stop a drug	29	3.2	27	3.3	0.1800
Other	18	2.0	68	8.3	<0.0001^a^
Total number of patients	895		816		

Pearson *χ* ^2^ with Bonferroni adjustment 0.05/53 0.0009

a Statistical significant difference.

## Discussion

The data obtained in Phase 1 were derived from a large sample of patients, but a relatively small number of pharmacists in only four northern Italian cities, where the cooler temperatures may have an adverse effect on asthma control. In contrast, the findings from Phase 3 involved a similar number of patients but recruitment was spread over 15 out of 20 Italian regions, from the North to the much warmer South, and almost three times the number of pharmacists were involved. The similarities in the prevalence of inappropriate medicines used, such as ACE-I and *β*-blockers, perceptions of problems, adherence and smoking across the two studies, however, suggests that the results obtained in Phase 1 were confirmed in Phase 3. Furthermore, a total of 53 items were compared in this analysis, and only 15% (8/53) of them presented a statistically significant difference. Thus, these results suggest the need for an intervention, which could provide potential benefits for patients with asthma.

The finding that around 50% of patients were adherent to asthma treatment is in line with the results of a systematic review (Engelkes *et al*., 2015), which found that adult adherence rates to asthma treatment was between 30 and 70%. The relatively high rates of smoking in patients with asthma are reflective of the high national prevalence in Italy (21%) (Lugo *et al*., 2017).

The process measures recorded, PCIs identified and actions taken, during delivery of the intervention, were also consistent between the two phases. One possible reason for such consistency is that the patient population was similar, despite the broad inclusion criteria, but the use of a standardised, structured template and similar training could also contribute to these findings. Given that Phase 3 demonstrated the I-MUR to be both clinically effective and cost-effective, it is reasonable to anticipate that, provided the structures are consistent (training, private area, other pharmacist inclusion criteria met) the processes maintained through the use of a structured interview, the benefits demonstrated in Phase 3 should be achieved in a commissioned service.

The mean number of PCIs per patient of 2.5 and 2.4 in Phases 1 and 3, respectively, and the most common action taken by pharmacists being patient education required in both populations are similar to the studies in other European countries. A Danish study conducted in 2001 (Herborg *et al*., 2001) found that the most common DRP in asthmatic patients was poor knowledge of asthma, suggesting that, as was found here, patient education was required. A German study (Schulz *et al*., 2001) reported the most common advice given to asthmatic patients by pharmacists was drug information. This was also the most common recommendation in a study in England of patients with multi-morbidity (Krska *et al*., 2000), which used the same classification system for quantifying PCIs and pharmacist actions. Other studies in multi-morbid patients have, however, found a higher number of DRPs (Brulhart and Wermeille, 2011).

The lack of consistency in provision of an intervention found in some studies (Krska and Avery, 2008; Hinchliffe, 2011) is a potential problem for a commissioned service. Greater standardisation of the intervention, using the mechanisms employed here, may help to reduce such variation. Although it must be acknowledged that structured questioning is somewhat contrary to the open-ended approach to medication review advocated in England, which seeks to improve patient-centred consultations (Picton and Wright, 2013), the inclusion of selected standardised questions into the process and recording of these data could help to provide a greater degree of evidence, while still allowing for individualised care.

### Strengths and limitations

The data presented here involved 1711 patients and 275 pharmacists, thus the combined data represent one of the largest reports describing a pharmaceutical care intervention. The two studies, Phases 1 and 3, were conducted at different times, in different locations, by different pharmacists and with different patients, yet showed similar findings. The large sample sizes included also support the generalisability of the results achieved using I-MUR in Phase 3.

The data gathered during the I-MUR (medications used, problems, PCIs and actions) were uploaded onto a web platform by the pharmacists who conducted the I-MUR and it was not possible to verify the accuracy of these. The classification of PCIs and actions was dependent on the interpretation of the pharmacists and these were not validated. However, this approach did facilitate the conduct of two large-scale studies at minimal cost and no problems were encountered in use of these classification systems, despite the large number of pharmacists involved.

## Conclusions

A structured approach to medication review for asthma has the potential to improve outcomes due to consistency in delivery and there is potential to benefit from the I-MUR across Italy. It may be appropriate to consider using a similar structured approach for other conditions and in other countries. Involving pharmacists in gathering patient-level data may be a useful strategy to help generate the type of evidence suggested as being required to support CPS such as medication review.
